# Automatic Identification and Representation of the Cornea–Contact Lens Relationship Using AS-OCT Images

**DOI:** 10.3390/s19235087

**Published:** 2019-11-21

**Authors:** Pablo Cabaleiro, Joaquim de Moura, Jorge Novo, Pablo Charlón, Marcos Ortega

**Affiliations:** 1 Centro de investigación CITIC, Universidade da Coruña, 15071 A Coruña, Spain; pcc.sdc@gmail.com (P.C.); jnovo@udc.es (J.N.); mortega@udc.es (M.O.); 2 VARPA, Instituto de Investigación Biomédica de A Coruña (INIBIC), Universidade da Coruña, 15006 A Coruña, Spain; 3 Instituto Oftalmológico Victoria de Rojas, 15009 A Coruña, Spain; pablo@pchoptometria.com; 4 Hospital HM Rosaleda, 15701 Santiago de Compostela, Spain

**Keywords:** computer-aided diagnosis, optical coherence tomography, anterior segment, cornea, contact lens

## Abstract

The clinical study of the cornea–contact lens relationship is widely used in the process of adaptation of the scleral contact lens (SCL) to the ocular morphology of patients. In that sense, the measurement of the adjustment between the SCL and the cornea can be used to study the comfort or potential damage that the lens may produce in the eye. The current analysis procedure implies the manual inspection of optical coherence tomography of the anterior segment images (AS-OCT) by the clinical experts. This process presents several limitations such as the inability to obtain complex metrics, the inaccuracies of the manual measurements or the requirement of a time-consuming process by the expert in a tedious process, among others. This work proposes a fully-automatic methodology for the extraction of the areas of interest in the study of the cornea–contact lens relationship and the measurement of representative metrics that allow the clinicians to measure quantitatively the adjustment between the lens and the eye. In particular, three distance metrics are herein proposed: Vertical, normal to the tangent of the region of interest and by the nearest point. Moreover, the images are classified to characterize the analysis as belonging to the central cornea, peripheral cornea, limbus or sclera (regions where the inner layer of the lens has already joined the cornea). Finally, the methodology graphically presents the results of the identified segmentations using an intuitive visualization that facilitates the analysis and diagnosis of the patients by the clinical experts.

## 1. Introduction

Contact lenses are very present in the daily routine of many patients, their applications being broadly increased in the field of ocular medicine in the recent times. As a reference, they are typically used from cases of myopia or astigmatism to pathologies such as keratoconus, marginal pellucid degeneration, corneal transplants or cases of trauma in the ocular region that can produce irregularities in the cornea of patients [1].

These irregularities, for example, produce areas on the cornea that hinder the passage of light and, therefore, penalize the quality of vision. For the visual rehabilitation of these patients, solutions such as the use of mini-scleral, semi-scleral and scleral contact lenses (SCL) have arisen over the last years, which must present absence or reduction of the contact with the cornea [1,2]. These cases imply the necessity of studying both the ocular structure and the separation between the lens and the cornea over different instants of the rehabilitation process, selecting the different SCL curves to maintain a correct vault over all the cornea.

**Figure 1:**
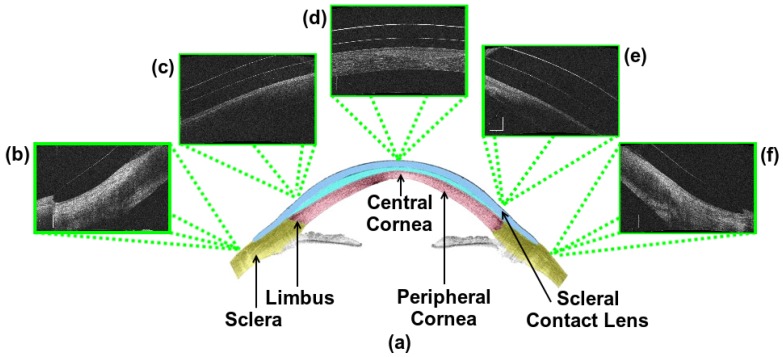
Schematic representation of anterior segment optical coherence tomography (AS-OCT) images. (**a**) An illustrative example of the anterior pole segment, including sclera, limbus, central cornea, peripheral cornea and scleral contact lens. (**b**,**f**) Extreme regions of the SCL where the outer layer is the one that meets the sclera. (**c**,**e**) Peripheral cornea and limbus region where the inner layers of the SCL progressively approach and finally join to the sclera. (**d**) Central cornea where the SCL always maintains a distance to the cornea.

For this purpose, new diagnostic imaging techniques are currently increasing in relevance, among which anterior segment optical coherence tomography (AS-OCT) images stands out. AS-OCT was developed in 1994 by Izatt et al. [3] and is today one of the most informative modalities used in the study of the anterior chamber or segment, the area in the anterior third of the eye from the back surface of the cornea to the crystalline lens. Its use does not require any contact, being non-invasive for the patient and producing images of both the anterior and posterior segments. These images allow the study of both the thickness and volume of the entire cornea as well as the biometry of the camera, having applications in contact lens (CL) fitting, diagnosis and clinical evaluation, surgical planning and monitoring of patients with many significative eye diseases [4]. Moreover, they allow the detection of micro-structural changes in the cornea of the patient, being very useful for studying the characteristics of the relationship between the CL and the cornea. However, the lack of automatic procedures requires manual study by the clinical experts, making the process extremely difficult and limiting the amount of information that is extracted from the images. Figure 1 shows representative examples of AS-OCT images that were obtained from different eye regions.

Taking into account the significant impact this capture technique has presented on the diagnostics and monitoring of diseases of the eye and the intravascular domain, developments are underway to expand OCT to other medical applications including gastroenterology, dermatology, dentistry and gynecology, among others. In this context, given the potential of this capture modality, over the recent years, different computational proposals have been presented related to the automatic segmentation of different biological and pathological structures over a disparity of OCT image modalities.

Regarding the processing of AS-OCT images, there are two major areas of application. The first implies the study of the angle of the anterior chamber [5,6], which presents its main application in the diagnosis of glaucoma [7,8,9]. On the other hand, we find methodologies that focus on the detection of the edges of the cornea in order to study its topology. As a reference, Graglia et al. [10] developed an algorithm to detect the center points of the anterior and posterior parts of the retina to continue with an analysis pixel by pixel detecting the contour of the cornea in images with strong and clear contrast. Williams et al. [11] developed a methodology for the segmentation of the entire anterior segment of the eye using shape priors levels sets on the resulting images from a thresholding process, obtaining comparable results with the manual segmentations of the experts. In a posterior study [12], the authors used graph cut techniques for the detection of the anterior segment of the eye, improving the accuracy of the results as well as accelerating the required computational times. Shen et al. [13] used a more direct approach based on the thresholding of the images that present difficulties in the detection of the posterior surface of the cornea. More complex approaches have been adapted using gaussian mixture models (GMM) [14] or the combination of GMMs with graph cuts and levels sets [15]. Other techniques such as graph theory and dynamic programming have also been used with spectral-domain images (SD-OCT) to analyze only the central region of the cornea [16]. Using the gradient and the edges of the image, this method employs the Dijkstra algorithm to find the shortest paths representing the epithelium, the Bowman layer and the corneal endothelium. This method obtains correct results in the central areas of the images since an easier analysis can be archived in these regions. More distant regions are estimated by a polynomial wave that uses as reference the detected surface. Approaches have also been developed in three dimensions [17], obtaining acceptable results in the central part of the cornea in studies that were done with mice.

While there exist different works in the literature about the analysis of AS-OCT images, to date there is no other methodology designed for the study of the relationship between the cornea and the SCL, as in our case. This work proposes a fully automated system for the extraction of the region of the SCL and the cornea using AS-OCT images in the analysis of the entire eye region. In particular, the study is performed not only in the typical central region but also in the most complex cases as the lateral and the extreme regions of the eye represent. Figure 1 presents representative examples of the three possible regions of analysis (central, lateral and extreme) as well as their corresponding general locations in the eye. This comprehensive approach implies a complex analysis with zones that present a significant variability in contrast and orientation. Then, using the obtained cornea and SCL segmentations, we calculate representative distance metrics that quantify their relationship. Specifically, we obtain three metrics: The vertical distance between the SCL and the cornea, the normal distance to the tangent of the points of both the SCL and the cornea and the closest distance point of the points of both the SCL and the cornea. These different metrics can be used to study the comfort or potential damage that the CL can produce in the eye, selecting the different curves of SCL to maintain a correct vault throughout the cornea. With this, we aim at demonstrating the usefulness of the automatic analysis of the cornea–CL relationship in AS-OCT images by obtaining complex metrics that are not feasible to measure by manual analysis. Additionally, the method builds and returns a clear and intuitive colored visualization system that facilitates the posterior analysis and diagnosis of the expert clinicians.

The manuscript is organized as follows: Section 2 presents the proposed methodology and the details of each of its phases; Section 3 presents the results that were obtained by the methodology and its validation using as reference the manual segmentation of an expert and, finally, Section 4 and Section 5 present the discussion and main conclusions of the work together with possible future lines of research.

## 2. Materials and Methods

The proposed methodology receives as input one or more AS-OCT image with the information of the biological tissues corresponding to the cornea and the SCL. Typically, these images correspond to the central cornea (Figure 1d) where the SCL always maintains a distance to the cornea, the peripheral cornea and the limbus (Figure 1c,e) where the inner layers of the SCL progressively approach and finally join the sclera or the region of the extreme of the SCL (Figure 1b,f) where the outer layer is the one that meets the sclera.

**Figure 2:**
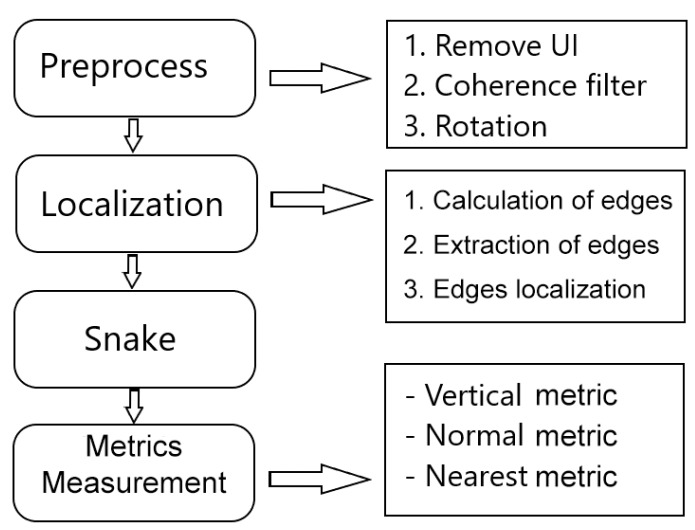
General scheme of the proposed methodology.

The method is composed by four sequential and progressive phases, as illustrated in Figure 2. First of all, given the limitations that these images usually present, a preprocessing of the image is performed to reduce the level of noise and increase the contrast. Next, we estimate the relative position of the image on the eye by studying the distribution of intensities along the image. Finally, we use active contour models, snakes, for the final adjustment and segmentation of the cornea and SCL regions.

In particular, active contour models represent an important and well-established segmentation paradigm that achieved satisfactory performances in many segmentation tasks due to its ability to obtain a larger convergence range as well as to handle natural topological changes. For that reason, different active contour models and their variants were successfully used for boundary segmentation in different medical imaging modalities. As reference, Deglint et al. [18] employed snakes for measuring the arterial wall diameter of astronauts from ultrasound images. Lui et al. [19] proposed a method for unique external energy applied to a slightly modified decoupled active contour (DAC). This method combines textural variation based on a sparse texture model with structural variation. In the work of Beevi et al. [20], a localized active contour model (LACM) was used for the segmentation of cells to exploit variation in cell size through the different phases of mitosis. Nithila et al. [21] presented a region based on an active contour model using a variational level set function (SPF) for segmentation of the lungs from CT images. On one hand, as we can observe, some of these works propose the use of active contour models in a way that they adapt to particular domains where the complexity of the region to the segment allows for the use of higher-level information such as texture. The particular domain for this work, where the contact lens region, for instance, does not possess high-level details, makes these approaches less suitable for the task. On the other hand, some of the models increase the computational requirements of the used algorithm, some even being semi-automatic methods developed for efficiency reasons which, also, is an undesirable property for our problem, given the necessity of offering an automated and fast tool for the specialist in order to make it usable in real-world scenarios.

In line with that, and in order to take a deeper look into a particularly similar domain to ours, it is noticeable that, recently, snakes were satisfactorily used for the segmentation of retinal layers in OCT images [22,23,24]. In particular, González-López et al. [24] proposed a strategy based on snakes for the segmentation of the main retinal layers where good results were obtained from the designed experiments, outperforming previous existing proposals in this specific domain, including a geometric level set formulation of an active contour model [25] and a deep learning approach [26], among others. Given that context, we considered that the characteristics of the snake fit better in the particular segmentations of our problem due to their simplicity of implementation, robustness and efficiency in terms of required computational resources.

Using these segmentations as reference, we proceed to a phase of calculating the mentioned metrics and construct the final visualization for the analysis of the specialist. Given that there are numerous parameters in these steps and in order to search for the optimal configuration of the system, we finally use an evolutionary algorithm for the parametric optimization of the proposed method.

### 2.1. AS-OCT Image Acquisition

**Figure 3:**
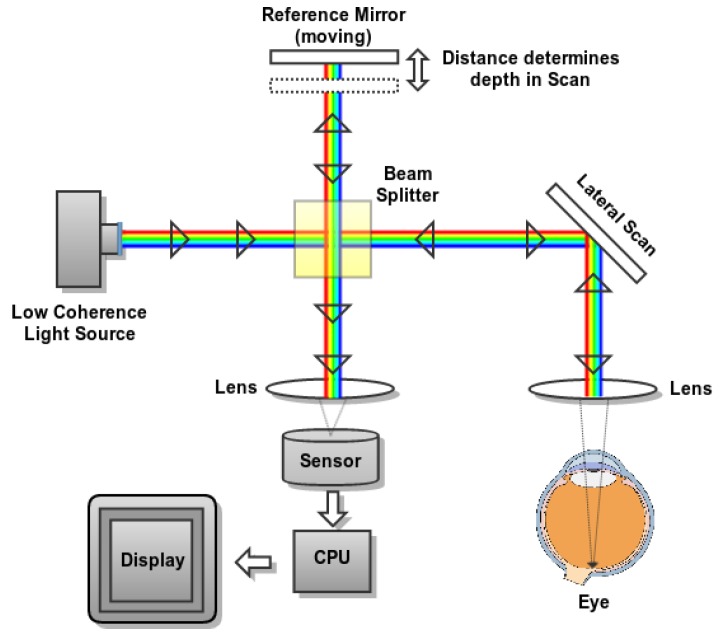
Schema of a basic optical coherence tomography (OCT) acquisition system.

AS-OCT is based on the optical coherence tomography (OCT) capture technique, a widely used non-invasive imaging technology that provides high-detail transverse images (tomographies) of the main internal structures of the biological tissues [27,28,29]. Figure 3 illustrates a typical optical configuration of a basic OCT acquisition system. To obtain the OCT image, the instrument scans a beam of light laterally, creating a series of axial scans with information on the intensity of the reflected signal as a function of depth [30,31]. In particular, the OCT technology is based on Michelson interferometer, which is a configuration commonly used in optical interferometry. This configuration includes two mirrors, one of which is, in this case, the ocular tissue samples and the other serves as a reference mirror. The light reflected from the ocular tissue samples is combined with the light reflected from the reference mirror, producing a set of cross-sectional OCT scans.

### 2.2. Preprocessing Stage

The three main limitations that are typically encountered in AS-OCT images are the characteristic levels of noise that are present in the images, the low contrast of the background and the tissues and the fact that the images can be from approximately horizontal (central region) to significantly diagonal (lateral and extreme regions). For these regions, we designed a preprocessing stage with the objective of simultaneously reducing the levels of noise as well as improving the contrast in order to facilitate the posterior stages of the proposal.

**Figure 4:**
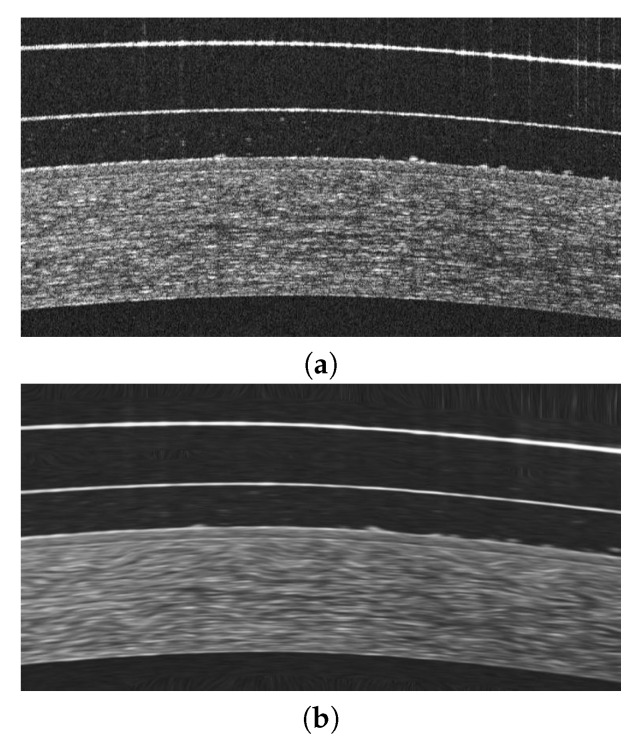
Example of application of the non-linear anisotropic diffusion filter. (**a**) Optical coherence tomography of the anterior segment (AS-OCT) image of the central region. (**b**) Result after the filtering process.

As a solution for these purposes, we propose the use of a non-linear anisotropic diffusion filter [32] using an implementation of Kroon and Slump [33] and a non-negative discretization. Given that the subsequent analysis will focus on the study of the edges of the image, the reason for using this filter lies in its ability to eliminate as much background noise as possible without blurring these edges, simultaneously increasing its enhancement and eliminating possible internal gaps. A representative example of the result after applying this filter can be seen in Figure 4.

The fact that the input images can present different positions (previously illustrated in Figure 1) is problematic for the proposal of a general methodology that automatically handles all the possible scenarios adequately. The analysis of the central images, given the horizontal disposition of the target objects and tissues, can be optimized by the direct inspection of the images by rows and columns. However, the analysis of the lateral and extreme images supposes a higher computational cost for the detection of the same regions of interest. To improve this, we rotate all the images to present the most possible horizontal distribution of the tissues and SCL and, therefore, facilitate the subsequent analysis. In particular, we rotate the lateral and extreme images, where the predominant direction of its edges is usually diagonal until we reach a result where we have most of the edges in an approximately horizontal position.

**Figure 5:**
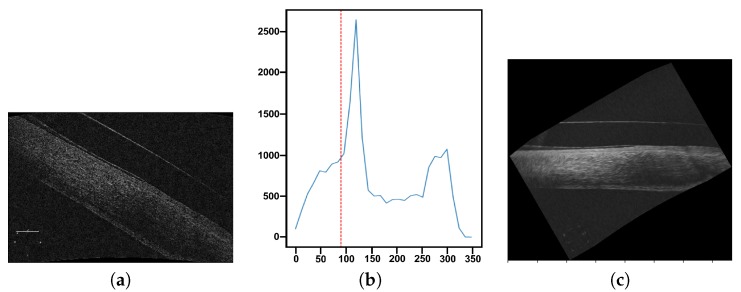
Example of the filtering and rotation process of the preprocessing stage. (**a**) AS-OCT image of a lateral region to be preprocessed. (**b**) Representation of the calculated histogram of oriented gradients (HOGs) where the discontinuous red line marks the majority position that we characterize as horizontal. (**c**) Result of applying the non-linear anisotropic diffusion filter, rotation and the top hat filtering to the image in order to produce an enhanced image with the maximum number of edges with a horizontal distribution.

In particular, to rotate the images, we use the histogram of oriented gradients (HOGs) [34] in order to study the distribution of the directions of the edges in the image. In this way, we obtain an approximate representation of which directions the edges point to, as can be seen in the illustrative example of Figure 5c. Using the HOG information, we rotate the image in such a way that as many edges as possible present a horizontal direction and vertically oriented gradients within the image. Finally, we enhanced the image using the top hat algorithm in order to be able to obtain more representative information from the edges in the next phases. A final representative result of this entire process is presented in Figure 5c.

### 2.3. Preliminar Localization of the Cornea and the Scleral Contact Lens

In this phase, we look for an approximation of the target regions to be segmented in the cornea and the SCL. This preliminary localization posteriorly serves as an initialization of an active contour for the final precise segmentation. Specifically, the objective of this phase is to obtain an approximation of the external and the internal layers of the SCL and the cornea. To do this, the method performs three consecutive steps: Extraction of the edges using the Canny algorithm [35], an estimation of the areas of interest and a pixel to pixel border approximation.

#### 2.3.1. Identification of the Initial Searching Rows

**Figure 6:**
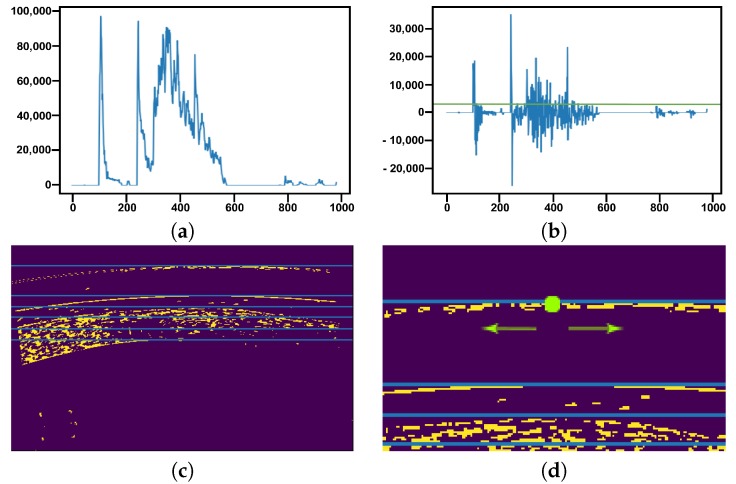
Example of the localization of the initial searching rows. (**a**) Distribution of intensities along the analyzed rows of the image. (**b**) First order derivative of (**a**) using a threshold that discriminates the points to be studied. (**c**) Result of applying Canny to the preprocessed image and the identified initial searching rows. (**d**) Initial point of the outer layer (in green) detected from the initial searching rows and from where the extraction of the preliminary points process begins.

For the estimation of the areas of interest, we take advantage of the approximately horizontal disposition of the images after the preprocessing phase (Figure 5c). With this in mind, we can perform a brief study of the image based on the distribution of the intensities between rows, as presented in the example of Figure 6a. This analysis is performed over the edges of the image, as we can assume that the limits of the layers of the cornea and the SCL are the areas with the higher number of gradients. Using the first order derivative, we can identify those points with accumulations of edges, that is, the limits of the target layers. Using a threshold over this derivative, as illustrated in Figure 6b, we can determine the most salient variations. An example of the final identifications of this process, the initial searching rows for the following steps, can be seen in Figure 6c.

**Figure 7:**
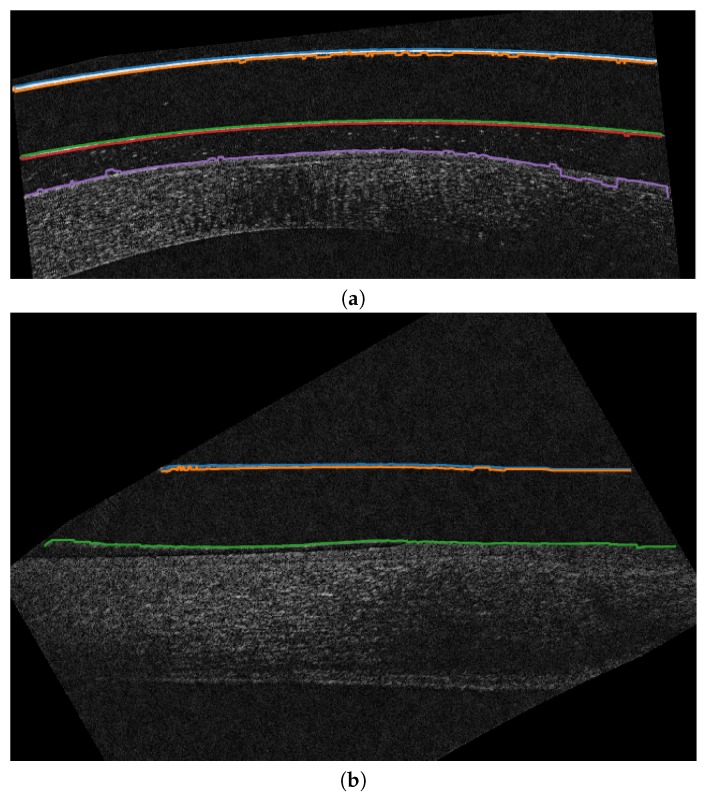
Example of localization of the areas of interest. (**a**) AS-OCT image of the central region with the outer and the inner layers of the scleral contact lens (SCL) and the cornea localized. (**b**) AS-OCT image of the lateral region with the outer layer and the cornea localized.

To differentiate the cases where we only find the outer layer of the SCL and the cornea (lateral cases), only the cornea (extreme cases) and the cases where the three layers are present, we always look for the first three areas of interest that we approximate and that present an acceptable quality. With these three potential initial rows for the target layers, we studied the intensity profiles over these regions to effectively identify them. The edges from the SCL should present a slight intensity difference because the dark background is maintained while the cornea is more visible given that its tissue is captured with a very differentiable intensity within the image. Once the differences are studied, the one with the most significant variations is marked as the initial row for searching the cornea, obtaining results like those presented in Figure 7.

#### 2.3.2. Extraction of the Preliminary Points of Each Target Regions

Once the initial searching rows are obtained (Figure 6c), for the correct extraction of the target regions we must minimize the impact of the noisy artifacts that appear near to the layers, the gaps in the edges and the progressive loss of contrast as we get closer to the extremes of the images.

We adjust this result to the layer morphology looking for edges close and below the estimated initial row detecting a point that we assume to belong to the outer limit of the outer layer of the SCL. Using this point as reference, we study the points in its neighborhood that belong to the same edge and could belong to the same layer. We repeat this procedure adding one pixel at time to a list of points that we assume to be the outer limit of the outer layer of the SCL. Since this process can be very complex due to the noise that is still present in the images, we use a set of criteria in order to find the points that could belong to the target of this step:

- If a point does not have enough points in its neighborhood with a significant intensity, it is considered a possible artifact.
- If a point has several neighborhood points that could be the continuation of the edge, the point that we are looking for is the one that maximizes the smoothness of the layers. To do this, we look for the point that is closer to the theoretical point that is estimated using the orientation of the previous points.
- In case no point in the neighborhood meets the previous criteria, the edge keeps going in the orientation of the previous points. In case of being a gap, once we find the other extreme of it, we correct the edge interpolating the points in the existing gap.

Once we have the outer limit of the outer layer, we look for the inner limit of this layer. To do this, we use the first derivative of the distribution of intensities along the image (Figure 6b) looking for negative changes in intensity below each point of the layer that was found previously. In case we find gaps or zones without significant intensities, we follow the outer limit maintaining the layer width found in the previous points.

We repeat this process on each detected layer and, as result, we obtain an approximation of the segmentation of the three lines of interest: The outer layer of the SCL, the inner layer of the SCL (if it is not the same as the cornea already) and the cornea.

### 2.4. Precise Segmentation of the Cornea and the Scleral Contact Lens

**Figure 8:**
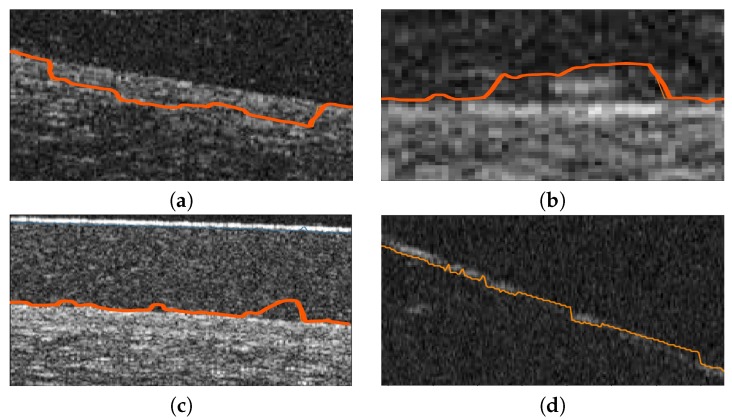
Example of results of the preliminary localization stage prior to the precise segmentation in different AS-OCT images (**a**–**d**).

Using as reference the preliminary localization of the previous phases, the method precisely segments each target layer. Given the significant noise that is typically present in these images, the identification of clear and smooth lines for both surfaces of the SCL and the cornea constitutes a complex issue. Moreover, even after the preprocessing stage, preliminary localization usually presents sudden jumps far from the target edges given the presence of artifacts or low contrast of the image in certain areas. Representative examples of these problems can be found in Figure 8.

Given these drawbacks and the fact that we want to extract coherent and smooth segmentations, we employ an active model, in particular, a snake [36], over the preliminary localization to improve our detection of the inner layer of the SCL and the cornea. In particular, snakes were used for the segmentation of boundaries in different medical imaging modalities demonstrating the suitability of this strategy in terms of simplicity of implementation, robustness and efficiency of required computational resources. In particular, as representative examples, similar approaches were satisfactorily applied in OCT, a very related domain about the analysis of the eye fundus, for the segmentation of the retinal layers [22,24,37] or the measurement of the retinal thickness [23], among other applications. A snake consists of a deformable model under the influence of internal forces that restrict the shape of the model (for example, its smoothness) and external forces that push the curve towards characteristics that are present in the image (for example, the edges). The energy of the snake depends both on its shape and its position in the image, so we try to find the local minima of the energy function, representing the properties of the objects that we want to detect.

We can represent the position of the snake parametrically as v(s)=(x(s),y(s)) such that s=ε[0,1] gives a solution to the snake. With this equation, the search of the configuration where the compromise between internal and external forces is optimal can be considered as a minimization problem with the following form:

(1) $$\varepsilon \left(v\right)=\varepsilon_{int}\left(v\right)+\varepsilon_{ext}\left(v\right)$$

where $\varepsilon_{int}\left(v\right)$ measures the internal energy and $\varepsilon_{ext}\left(v\right)$ represents the external energy. The internal energy depends on the first and the second order derivatives, respectively, representing the elasticity and the rigidity of the curve. We can formulate this energy as follows:

(2) $$\varepsilon_{int}\left(v\right)=\int_{0}^{1}\alpha \left(s\right)|v_{s}{\left(s\right)|}^{2}+\beta \left(s\right){\left|v_{ss}\left(s\right)\right|}^{2}ds$$

Variables α and β are the constants that are in charge of controlling the shape to which the curve tends. That is, they constitute the mechanism through which we can define how soft or how flexible the curve can be.

On the other hand, the external energy depends on the intensity characteristics of the image, such as edges or intensity profiles. We will abstract these characteristics as the potential of the image P(v), in such a way that we can represent the external energy as:

(3) $$\varepsilon_{ext}\left(v\right)=\int_{0}^{1}P\left(v\left(s\right)\right)ds$$

From this formulation, a snake progresses iteratively evaluating each point and, in its neighborhood, the energy function, placing the point in the position of the neighborhood with the lowest energy value. This process is repeated until the model reaches a convergence or a maximum number of iterations.

**Figure 9:**
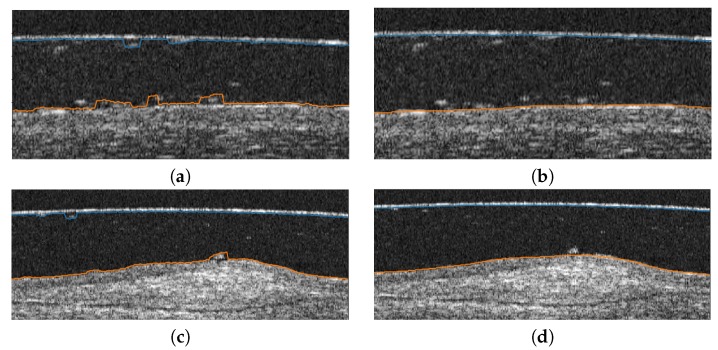
Example of the results of the segmentation phase in different AS-OCT images. (**a**,**c**) Preliminary localizations. (**b**,**d**) Result after applying the snake to the (**a**,**c**) images.

For this phase, we use the implementation of a snake as a “free snake” for the extreme points, allowing the progressive movement of these points through the iterations. Given the mentioned problems we faced in the preliminary localizations with significant irregularities due to background noise or other artifacts, we boost the smoothness of the snake with high values for the parameter β (Equation (2)) in order to overcome these irregularities. In particular, this increase the rigidity of the curve producing a significant smoothness that avoids sharp corners or changes. Illustrative examples of the results of this phase can be seen in Figure 9.

### 2.5. Calculation of the Distance Metrics

Once we produce the final segmentations of the cornea and the inner layer of the SCL, we can measure and study their relationship in terms of distances. To study this relationship, it is necessary to firstly define the distance metric that is used between the corresponding points from the SCL and the cornea. In our case, we use the Euclidean distance for any two points p and q, presenting the following form in the case of n dimensions:

(4) $$ED\left(p,q\right)=\sqrt{\sum_{i=1}^{n}{\left(q_{i}-p_{i}\right)}^{2}}$$

Additionally, we need to define the correspondence of the points from each layer, that is, from any point of the cornea and the SCL, and identify its corresponding point in the other region to measure their distance. In this work, we have defined three alternative paradigms for the selection of the corresponding points: Search in the vertical line of each point; search in the normal line of the tangent of each point; and, finally, the identification of the nearest point.

#### 2.5.1. Vertical Line Paradigm

**Figure 10:**
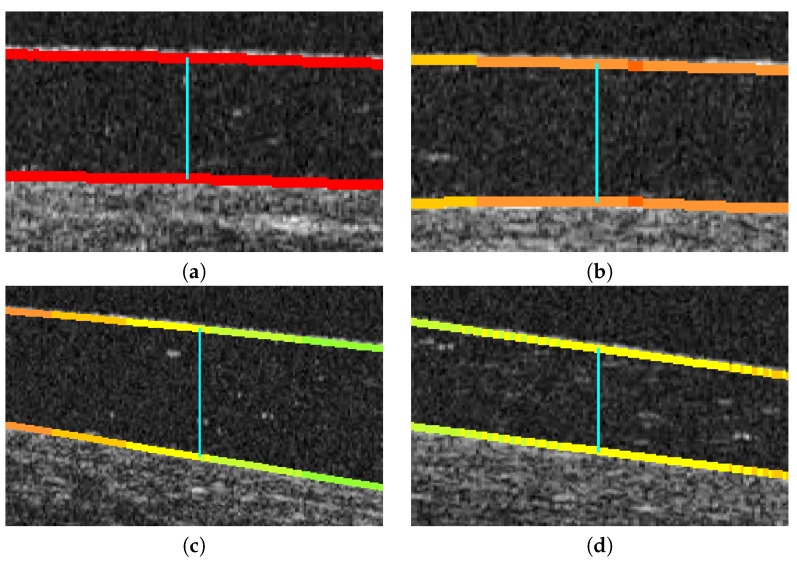
Illustration of the main limitation of the vertical line paradigm for the calculation of the distance between the cornea and the SCL. (**a**,**b**) Examples of correct applications of the vertical line metric. (**c**,**d**) Examples of the limitations of the vertical line paradigm in the case of leaning regions of the image, where the vertical distance does not accurately measure the cornea–CL relationship.

In this case, the implementation of the search in the vertical line is fast and direct. For each point, its corresponding position in the other region is found in the same column, and the Euclidean distance between them is directly calculated (Figure 10a,b). While this approach is the faster and simpler one, it can lead to significative errors in the case of leaning regions where the vertical distance does not accurately measure the cornea–CL relationship. This limitation can be seen in the examples of Figure 10c,d. As an improvement, but less efficient, we introduce the other two alternative paradigms.

#### 2.5.2. Normal Direction Paradigm

**Figure 11:**
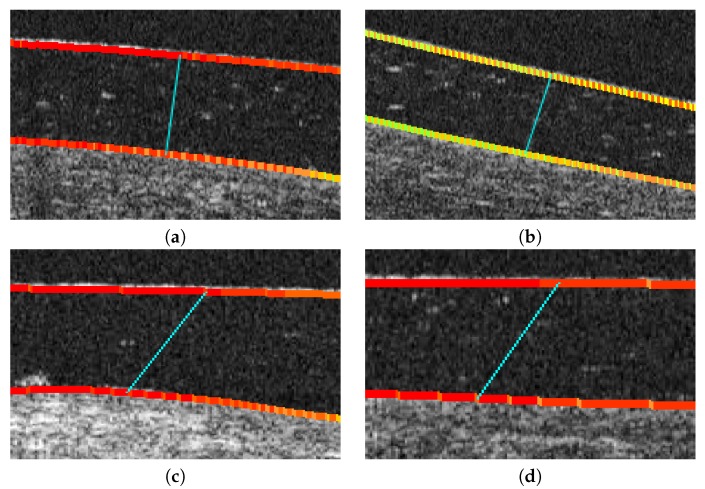
The main limitation of the normal direction paradigm for the calculation of the distance between the cornea and the SCL. (**a**,**b**) Correction of the normal direction paradigm to the problem of the vertical line metric examples of Figure 10c,d. (**c**,**d**) Examples of the limitation of the normal direction paradigm to sudden changes in the contour of the regions, where the normal distance does not accurately measure the cornea–CL relationship.

As said, this alternative paradigm is motivated by the previous limitation of the vertical approach. It is much more representative of the distances despite being more computationally expensive. In particular, for each analyzed point, the method determines the normal direction of the segmentation (using a neighborhood window of 10 pixels) and measures the distance until reaching a point on the opposite analyzed region (Figure 11a,b). This approach, despite being more robust than the vertical one, still presents some limitations when slight irregularities can remain in the extracted segmentations. These irregularities can provoke anomalous identifications of the normal direction providing imperfect distance measurements, as shown in the illustrative examples of Figure 11c,d.

#### 2.5.3. Nearest Point Paradigm

**Figure 12:**
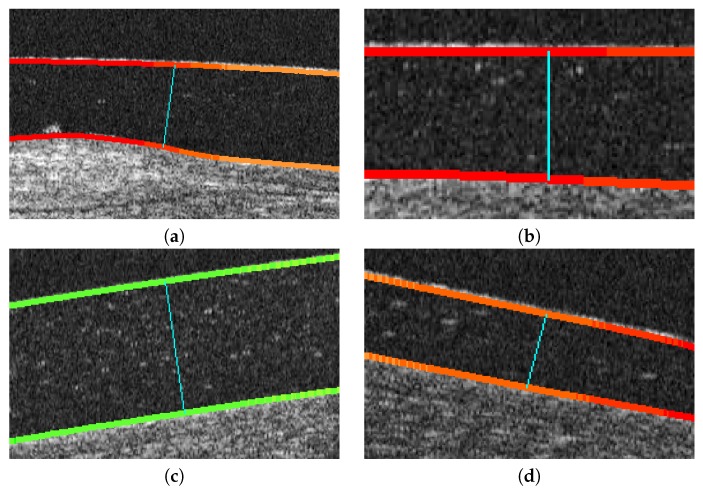
Examples of application of the nearest point paradigm for the calculation of the distance between the cornea and the SCL. (**a**,**b**) Solution of the nearest point paradigm to the limitations of the normal direction one in Figure 11c,d. (**c**,**d**) Additional illustrative examples of the nearest point paradigm, where the cornea–contact lens (CL) relationship is measured accurately.

**Figure 13:**
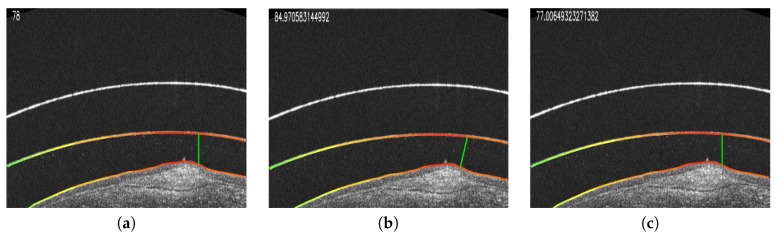
Examples of the cornea–CL relationship using the three designed paradigms for a selected point. The quantitative results are presented in the upper left corner of each example. (**a**) Vertical line. (**b**) Normal direction. (**c**) Nearest point.

To solve the problematic cases, we introduce a third approach that consists of an accurate search for the closest point of the opposite layer. In order to avoid expensive computational costs, we restrict to a discrete segment of points of the opposite region in the vertical projection as a local interval where we can assume the nearest point must be present and give the consistent and significantly smooth regions under analysis. In particular, we initially discretized this interval in a subset of points that are equidistantly distributed, identifying the nearest one. Finally, we intensively analyze in the neighborhood of the identified point pixel by pixel until we find the closest of all of them, which is finally returned as the nearest one. This approach, although presenting a higher complexity, is the most robust and representative since it characterizes the relationship between layers with a more precise minimum distance measure. Representative examples of the application of this paradigm can be found in Figure 12. In summary, an example of a result obtained by applying the three designed metrics for the same point can be jointly seen in Figure 13.

#### 2.5.4. Intuitive Heatmap Visualization

Using the designed paradigms, all the distances for the points of the inner layer of the contact lens and the cornea are measured, obtaining a list of distance measurements for all the target regions of the image. For their visual inspection, we built a final heat map using the distances that were calculated by each one of the metrics. In particular, for a better visualization, we mark as red the areas where the distance is minimal, whereas we mark as green the most distant ones, being the rest of the metrics proportional to the color scale. Additionally, the expert clinician can select any of the identified points of the resulting presented image to see the specific calculated distances using each approach, facilitating the analysis of the specialist. This presented information by selecting a point can be seen in the example of Figure 13.

### 2.6. Evolutionary Parametric Optimization of the Method

In the explained phases of the methodology, we used a series of parameters for its configuration that drastically determine its performance. Therefore, this method includes a significative set of possible configurations that enormously harden the identification of the optimal configuration by traditional or manual methods.

As an adequate way of finding the optimal configuration of these parameters, we selected the use of one of the evolutionary algorithms with greater potential and extensive use at present, differential evolution (DE) [38], which already demonstrated its utility in medical image analysis [39,40]. DE is a stochastic and population-based optimization method that seeks to improve a candidate solution from a measure of quality and through numerous iterations.

**Figure 14:**
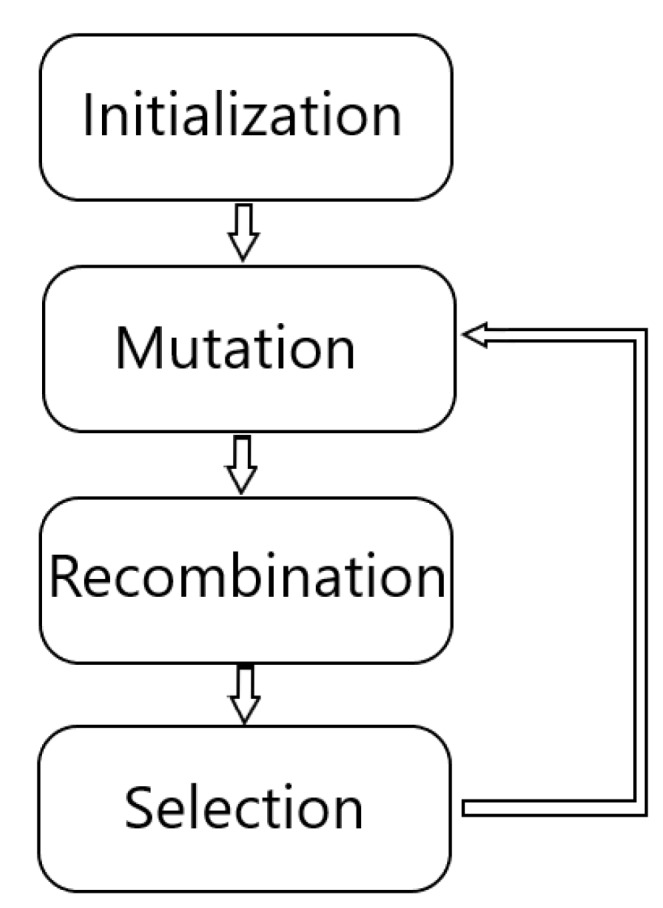
Phases of differential evolution for the parametric optimization of the proposed method.

DE consists of four main phases, three of them being iterative, as can be seen in the scheme of Figure 14. The phases consist of the initialization, mutation, recombination and selection [41]. In the beginning, with the initialization, we start from a set of D parameters, that constitutes the genotype, for each generation G of the population as follows:

$$x_{i,G}=\left[x_{1,i,G},x_{2,i,G},\ldots x_{D,i,G}\right],i=1,2,...,N.$$

where N is the size of the population, and must always be greater than four, and each parameter has an upper and lower limit $x_{j}^{L}\leq x_{j,i,1}\leq x_{j}^{U}$ from which the algorithm selects each one of the parameters uniformly in the interval $\left[x_{j}^{L},x_{j}^{U}\right]$. This process generates a set of N individuals *x*, each individual being defined as a set of possible values for each one of the D parameters; that is, the configuration parameters that we want to optimize.

In the mutation phase, for each individual $x_{i,G}$, three individuals $x_{r1,G}$, $x_{r2,G}$ and $x_{r3,G}$ are randomly selected in such a way that r1, r2 and r3 must be different. From this, we calculate the weighted difference of two of the individuals over the third:

$$v_{i,G+1}=x_{r1,G}+F\left(x_{r2,G}-x_{r3,G}\right)$$

where $v_{i,G+1}$ is the donor vector and F is the mutation factor, a constant in the range [0,2]. The objective of this phase is to expand the search spectrum by randomly generating new individuals; that is, new configurations for the method that iteratively converge over the subsequent generations to the optimal configuration set.

The next recombination phase introduces successful solutions from previous generations to the donor vector, generating the test vector $u_{i,G+1}$. To do this, the method makes use of a defined CR probability value, a random number obtaining function ${rand}_{i,j}∼U\left[0,1\right]$ and a value $I_{rand}$ to ensure that $v_{i,G+1}\neq x_{i,G}$ in the following way:

$$u_{i,G+1}=\left\{\begin{array}{c} v_{i,G+1}\;si\;rand_{i,j}\leq CR\;or\;j=I_{rand}\\ x_{i,G}\;si\;rand_{i,j}>CR\;and\;j\neq I_{rand} \end{array}\right.$$

where i=1,2,…,N, j=1,2,…,D.

In the selection phase, the target vector $x_{i,G}$ is compared to the test vector $u_{i,G+1}$. Finally, only the lower function value is allowed for the next generation in the following way:

$$x_{i,G+1}=\left\{\begin{array}{c} u_{i,G+1}\;si\;f\left(u_{i,G+1}\right)\leq f\left(x_{i,G}\right)\\ x_{i,G}\;in\;the\;other\;case. \end{array}\right.i=1,2,\ldots ,N$$

Thus, it would return to the mutation phase constantly until reaching some of the defined stop criteria.

Through this evolutionary paradigm, we identified the optimal configuration of the parameters of the previous phases, as said, given the high dimensionality of this problem that made it difficult to adjust them by other more traditional methods. For reasons of efficiency, we have discretized the search space of each parameter in a defined range in order to reduce the required computational cost. This search was performed on a subset of 56 images from the total of 112 images to avoid results that have arisen from over adjustments to a specific set of images, separating the other 56 as a validation set to obtain the final metrics of the proposed method.

## 3. Results

We conducted different experiments to validate the suitability of the proposed method using the public Contact Lens AS-OCT understanding (CLOUD) dataset [42] that was collected for this work and that contains 112 AS-OCT images that were captured from 16 different patients. From these, half were used for the configuration process, whereas the remaining 56 were reserved for the validation of the proposed method. In particular, the images were obtained by an OCT Cirrus 500 scanner model of Carl Zeiss Meditec with an anterior segment module for users of SCL. The studies were carried out with images from both eyes. The study adhered to the tenets of the Declaration of Helsinki and written informed consent was given by all participants.

For the validation, manual labeling of the inner layer of the SCL and the cornea was carried out by an expert for the validation of the results that were obtained in the segmentation phase. Additionally, the images were classified by the expert in the general defined groups: Central cornea images of the eye (the SCL and the cornea never touch), lateral regions (peripheral cornea, limbus and the beginning of the SCL scleral landing zone) and extreme regions (limbus and full scleral landing zone). This way, we were able to analyze the performance of the method in the segmentation of the cornea and the SCL in all the relative regions of the AS that can be represented in the AS-OCT images. Moreover, this is also helpful for obtaining the performance results about how the system is capable of correctly identifying the number of layers in all the scenarios: Central or lateral images (three layers present) and extremes images (two layers present).

Therefore, we studied the results with two objectives to measure: The segmentation adjustment to the regions of interest and to test if the method correctly classifies the images as having two (extremes images) or three layers (central and lateral images with the two layers of the SCL and the corresponding one in the cornea). Regarding the segmentation adjustment, for each point that was marked by the specialist, we measured the distance to the corresponding one in the column that was indicated by the method. This measurement is performed in all the points that were marked both for the cornea and SCL, obtaining finally the root of the mean squared error (RMSE) and the mean absolute error (MAE). In particular, the RMSE is defined as:

$$\mathrm{RMSE}=\sqrt{1/n\sum_{i=1}^{n}{\left(Y_{i}-{\hat{Y}}_{i}\right)}^{2}}$$

Moreover, the MAE is defined as:

$$\mathrm{MAE}=1/n\sum_{i=1}^{n}\left|Y_{i}-{\hat{Y}}_{i}\right|$$

Regarding the parametric optimization process, we used the implemented evolutionary DE with a population of 150 individuals, reaching the final optimal configuration values of Table 1 after 200 iterations. In particular, the optimization was performed in the parameters that control the median filter, the pixel search window neighbor both up and down in the pre-locating phase, the α and β parameters of the snake as well as the weighting term of the external energy, the limits for the Canny algorithm of the edges and the size of the kernel that it uses and, finally, the kernel size of the top hat algorithm for the enhancement of the image.

To avoid optimistic or skewed results, we additionally tested the performance of the method using the 56 images of the test set. The results of these analyses can be seen in Table 2 and Table 3.

**Figure 15:**
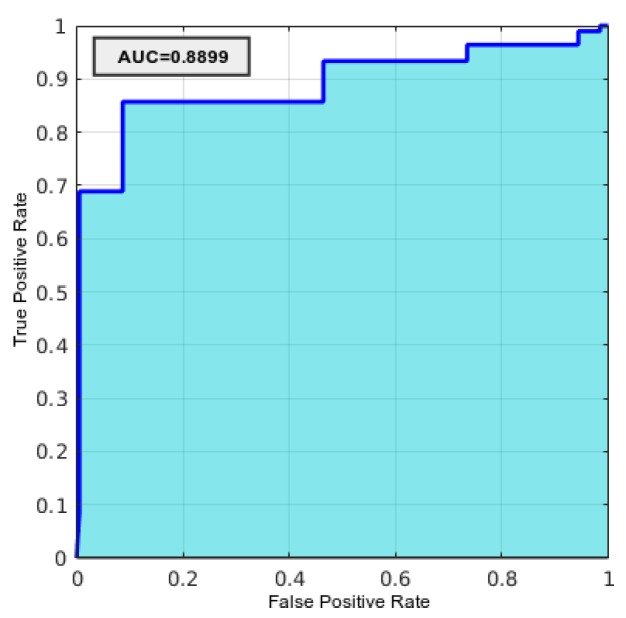
ROC curve of the classification results using the test set.

For the validation of the classification task, we defined a true positive as the correct detection of an image presenting two or less layers and a false positive as the correct detection of an image having three layers. With this in mind, in Table 2 we can see that the specificity, which targets the correct detection of cases that have two or fewer layers, is satisfactory. However, from the negative predictive value, we can see that there are some remaining cases where there are false negatives, that is, images that have the three layers but are classified incorrectly. This assessment can also be corroborated with the sensitivity, the percentage of positive images correctly classified, which shows that there are images with the three layers that are not correctly categorized. Moreover, Figure 15 shows the ROC curve obtained by the proposed system, with an area under the curve (AUC) of 0.889, reinforcing the validity of the designed methodology.

Regarding the segmentation performance that we can observe in Table 3, as we supposed, the method reached the highest values in central images, as they constitute the regions with a clear definition and contrast. These results are lower in lateral positions as the image quality is penalized with a lower representation detail of the tissues and a reduced contrast (as previously illustrated in Figure 1).

Regarding the computational costs, the average execution times of each phase can be seen in Table 4. Most of the computational load is concentrated in the phase of the segmentation using the snake, necessary for the elimination of artifacts and to obtain a smoother result with respect to the preliminary localization.

## 4. Discussion

The precise segmentation of the cornea and SCL using AS-OCT images allows the objective and precise study of the relationship between these regions of interest. This paper proposes a novel fully automatic methodology for the extraction and analysis of these regions, deriving representative metrics about their relationship and presenting the results in useful and intuitive heatmaps that facilitate the work of inspection and analysis of the specialists.

Regarding the results, we tested the proposed method with the Contact Lens AS-OCT understanding (CLOUD) dataset [42]. This public dataset can cover the central, lateral or extreme region of the SCL, presenting each scenario with particular complex image characteristics that are all coherently and automatically covered by the method.

Regarding the segmentation tasks, the use of snakes carries important advantages, as simplicity and computational efficiency, and desirable characteristics in many medical imaging applications. Moreover, genetic algorithms for parameter estimation also provided robustness in this adjustment. Additionally, it is important to take into account that, in other approaches, the segmentation using an active contour based model different from explicit snakes (such as level set-based approaches) is usually dependent on the placement of the initial contour, especially in complicated or heterogeneous samples. Sometimes even different results are obtained over the same image in a given segmentation task, depending on the use of different initial contours. Moreover, these models may become time-consuming if the periodical re-initialization step is adopted [43,44]. Given that context, we considered that the characteristics of the snake fit better in the particular segmentations of our problem.

Other proposals for the analysis of AS-OCT images were focused on the morphological study of the corneal region. However, to date, no other work faced the analysis of the cornea–CL relationship. In this case, we have to consider that the CL identification represents a challenging issue. Moreover, given the nature of the images with a large amount of noise and with different positions within the eye, it is often complicated even for the expert to classify and mark the images accurately, especially in the lateral and extreme areas of the eye where there is a significative deficit of information. Additionally, we would like to remark that all the developed source code is publicly available in the GitHub repository https://github.com/PabloCabaleiro/ProjectEyeliner to facilitate reproducibility and replicability at the hands of other researchers.

Given that no other extraction methodology of the cornea–CL relationship with AS-OCT images has been published to date, we have performed the validation based on the opinion of an expert to identify the region of interest and to be able to measure the adjustment of the segmentation results to it. In this way, we validated both the adjustment to the areas of interest and the classification of the image according to whether it has both layers of the SCL or not.

**Figure 16:**
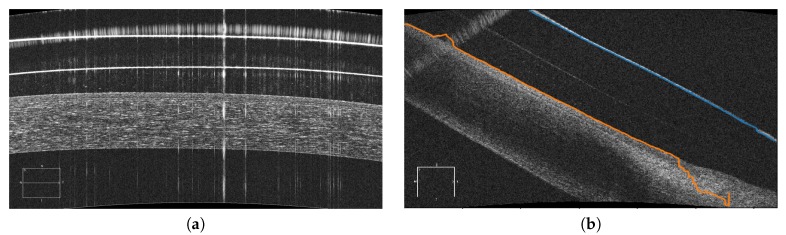
Examples of difficulties in the analysis of AS-OCT images. (**a**) Image with a high level of noise that hinders the normal processing. (**b**) Case where the inner line of the SCL does not present enough contrast.

This methodology presents an accuracy of 0.8235, which we consider a satisfactory result. We also obtained correct results in the adjustment over the different layers when the information that is present in the image is sufficient for this methodology. In cases where there is still noise such as the example in Figure 16a, the preliminary localization presents difficulties to obtain a successful adjustment and hinder the following phases. Most of these cases are caused by the amount of noise that is present in the images of this type and the low contrast of the areas of interest. This problem makes the preliminary localization of all the layers extremely difficult and, consequently, the classification of the images and the precision of the final segmentation of the aimed regions of interest (Figure 16b).

Finally, we present a visualization tool to facilitate the inspections of the results and subsequent diagnosis for the specialist, reducing the time that is needed to analyze AS-OCT images. With this tool, the specialist can access a new level of information about the relationship between the cornea and the SCL, quantifying this relationship, which improves the clinical analysis by the subjective visual inspection by the specialist.

We demonstrated the possibility of using AS-OCT images as a means to characterize the cornea–CL relationship using three different metrics: Vertical line, normal direction and nearest point. We have studied each of these metrics with its limitations and advantages, highlighting the results of the metric provided by the closest point as the most robust one. Moreover, as shown in Table 5, not only is it the most robust, but it also obtains better computational time than the implementation made for the normal metric, being constituted as an adequate balance between efficacy and efficiency.

## 5. Conclusions

This work presents a novel and fully automatic methodology for the segmentation and measurement of the cornea–CL relationship using AS-OCT images. The computational analysis of this relationship offers a significative amount of information and the potential to introduce more complex quantitative metrics for the subjective visual evaluations of the experts, also reducing the analysis process as well as improving the SCL fitting procedure. Additionally, the system allows a greater degree of follow-up of SCL wearers in order to avoid eye injuries that may be produced by the cornea–CL relationship during the SCL lifespan. All the code developed in this work is publicly available on the repository https://github.com/PabloCabaleiro/ProjectEyeliner.

The proposed method covers all the processing of the input AS-OCT image, from the preprocessing phase and the segmentation of the layers of interest to the calculation and visualization of the cornea–CL distance metrics. The proposed method was validated using the Contact Lens AS-OCT understanding (CLOUD) dataset [42], which contains 112 AS-OCT images from 16 different patients, using 56 for the parametric optimization and the remaining 56 for the validation of the results. These images were labeled by an expert, labeling that was used as a reference to measure the performance of the method both for the classification and the identification of the visible layers as well as the precise segmentation of each one of them.

As future work, we plan the improvement of the different phases to obtain more robust results. Specifically, we will focus on the preliminary localization phase to eliminate cases where the system fails. At the methodological level, we will seek to expand the number of experts with respect to those who validate the results in order to reduce the factor of human failure and the subjectivity of the task within the validation results. On the other hand, we plan to use sections of AS-OCT images for each eye, which allows us to study this relationship in 3D.

## Figures and Tables

**Table 1:** Optimal values that were reached by the evolutionary differential evolution (DE) method and used in the validation stage.

Parameter	Value
Median filter value	7
Localization: Top window	7
Localization: bottom window	7
Snake: α	50
Snake: β	20
Snake: edge weight	1
Canny: upper limit	120
Canny: lower limit	90
Canny: kernel’s size	3
Top hat: kernel’s size	21

**Table 2:** Validation of the classification results using the test set.

Statistics	Results
Accuracy	0.8235
Sensitivity	0.75
Specificity	1.0
Positive predictive value	1.0
Negative predictive value	0.6250

**Table 3:** Validation of segmentation results using the test set.

Metrics	Central	Lateral	Extremes	Global
RMSE	33.0931	73.7472	126.3294	80.4958
MAE	12.4934	39.1548	82.7255	39.5425

**Table 4:** Execution times of each phase.

Phase	Time (s)
Preprocess	0.7170
Preliminary localization	0.4742
Segmentation	4.8644
Metrics measurement	0.2570

**Table 5:** Execution times of each designed metric.

Metric	Time (s)
Vertical	0.0011
Normal	0.2102
Nearest	0.0457
